# Influence of the Potential Carbon Sources for Field Denitrification Beds on Their Microbial Diversity and the Fate of Carbon and Nitrate

**DOI:** 10.3389/fmicb.2018.01313

**Published:** 2018-06-22

**Authors:** Victoria Grießmeier, Johannes Gescher

**Affiliations:** ^1^Department of Applied Biology, Institute for Applied Biosciences, Karlsruhe Institute of Technology, Karlsruhe, Germany; ^2^Institute for Biological Interfaces, Karlsruhe Institute of Technology, Karlsruhe, Germany

**Keywords:** field denitrification beds, denitrification, methanogenesis, eutrophication, wood chips, wood pellets, wheat straw

## Abstract

Nitrogen based eutrophication of ecosystems is a global problem that gains momentum through a growing global population. The water quality of nitrate or ammonium contaminated rivers and streams cannot always be amended in centralized waste water treatment plants. Field denitrification plants were suggested as a solution for a decentralized reduction of nitrate to dinitrogen. Here, stable and cheap organic carbon sources serve as carbon and electron source for a microbial community. Still, our knowledge on the impact of these organic carbon sources on the development and diversity of these cultures is sparse. Moreover, the stability of these denitrification plants at different nitrate loading rates especially in the higher concentration regime were not tested so far. In this study, we compare the fate of carbon and nitrogen as well as the microbial community of wood pellet (WP) (pressed sawdust), wheat straw, and wood chips (WC) based laboratory denitrification reactors. Our study reveals that the diversity and composition of the community is strongly dependent on the carbon source. The diversity decreased in the order WC, wheat straw, and WPs. The three reactor types were characterized by different nitrate reduction kinetics and were affected differently by high nitrate loading rates. While the nitrate reduction kinetics were negatively influenced by higher nitrate doses in the wheat straw reactors, WPs as carbon source sustained the opposite trend and WC lead to an overall slower but concentration independent nitrate reduction rate. Counterintuitively, the concentration of soluble organic carbon was highest in the WP reactors but methane emission was not detectable. This is corroborated by the microbial diversity data in which methanogenic species were highly underrepresented compared to the other two reactor types. In contrary, the methane emissions in the wheat straw and WC reactors were comparable to each other.

## Introduction

Microorganisms, especially bacteria participate in important steps of the terrestrial nitrogen cycle. Next to nitrogen fixation and nitrification one essential process is denitrification. In this part of the nitrogen cycle nitrate is reduced over several intermediates (NO_2_, NO, and N_2_O) to atmospheric nitrogen. Denitrification is necessary to prevent an imbalance in the nitrogen cycle in favor of nitrogen input. This imbalance is mainly caused by the industrial NH_4_^+^ production via the Haber–Bosch process, which leads to a nitrogen fixation of 9.7 × 10^12^ mol year^-1^ (Canfield et al., 2010), whereof a significant portion is introduced into the environment (Erisman et al., 2008). Since the mid-20th century, the anthropogenic introduction of reactive nitrogen into the environment through fertilizer increased enormously due to an increasing food and energy production for a continuously growing global population. The amounts of nitrogen compounds measured at the onset of the industrialization were ten times higher as inflows measured about the end of the nineteenth century (Bednarek et al., 2014) and NH_3_ or NO emission increased about fivefold since then (Erisman et al., 2008). Next to artificial fertilizer, mainly urea as a component of manure is used in the agricultural sector, which is a main contributor to water pollution. This exaggerated use of fertilizer endangers 40% of the surface and 30% of the ground water in the European Union (OECD, 2012). One consequence of high inputs of fertilizer is the spreading of nutrients (nitrate, ammonium, and phosphate) into fresh and ground water, which causes eutrophication and a loss of biodiversity (Vitousek et al., 1997).

Moreover, the increasing demand of land for agricultural production necessitates that agricultural fields have to exist in close proximity to preserved ecosystems like bogs or fens. Field denitrification beds are one solution for a decentralized treatment of drainage waters from agricultural fields since they can minimize the impact of intense agricultural usage on adjacent ecosystems. These decentralized wastewater treatment systems have to be characterized by low construction costs, long durability, and self-sufficiency (Schipper et al., 2010). Usually, a low cost and rather stable source of organic carbon is used as carbon and energy source that sustains a microbial community consisting partly out of denitrifying microorganisms. The whole system is below the surface and drainage water is dammed up so that the activity of the microorganisms can lead to a depletion of available oxygen and a subsequent nitrate elimination via denitrification. Agricultural drainage water may also contain ammonium, which would require a nitrification step. Still, compared to municipal sewage treatment plants an aerobic tank for nitrifying bacteria was in previous studies often not necessary, because of the manageable amounts of ammonium (Elgood et al., 2010; Warneke et al., 2011b). Nevertheless, Schipper et al. (2010) investigated different field sites of which one showed substantial amounts of NH_4_^+^ in the inflow water (Schipper et al., 2010). Hence, the treatment of some drainage waters might necessitate also the integration of a nitrification step prior to denitrification. So far, studies presumed that microbial denitrification is the predominant process and other NO_3_^-^ reduction processes like dissimilatory nitrate reduction to ammonium (DNRA) play an underrepresented role in these beds. Also, the anammox process (anaerobic ammonium oxidation) conducted by members of the Planctomycetales was observed less frequently (Warneke et al., 2011a).

Denitrification beds function due to the anaerobic degradation of organic matter. Hence, it is possible that other anaerobic processes like methanogenesis will also be catalyzed at least to some extent. Methane production will lead to a loss of electrons that could be used for denitrification and will affect the sustainability of a denitrification bed because methane is a greenhouse gas. Next to a methane production also the formation of N_2_O, a 300 times more potent greenhouse gas than CO_2_ (Rhode, 1990), is possible due to an incomplete denitrification process. Moreover, N_2_O could also be a side product of nitrification (Bremner and Blackmer, 1978). Hence, investigations regarding the exact microbial composition and the catalyzed processes within these denitrification beds are necessary to model and predict nitrate elimination and to analyze their overall sustainability. One assumption is that the microbial composition in these beds is strongly dependent on the carbon material and influences the nitrate elimination process and the formation of side products.

Several studies (Saliling et al., 2007; Cameron and Schipper, 2010; Warneke et al., 2011c) investigated different carbon sources to find the most efficient one. Next to wheat straw (Saliling et al., 2007) and wood sources like wood chips (WC) (soft- and hardwood) also maize cobs, green waste (Warneke et al., 2011c), and sawdust with different grain sizes (Cameron and Schipper, 2010) were analyzed for their nitrate removal rates (Addy et al., 2016). Maize cobs showed the best nitrate removal rates compared to the other substrates, but they went along with a higher leaching of total organic carbon (TOC) and NH_4_-N (Cameron and Schipper, 2010; Warneke et al., 2011c). Wood products instead seem to provide ideal conditions for denitrifying organisms with less byproducts like for example TOC and N_2_O (Warneke et al., 2011c), even if they inhabit a lower nitrate removal rate than maize cobs (Warneke et al., 2011c). Nevertheless, the exact microbial composition of denitrification beds was recently investigated for the first time in WC filled systems. In this study, we revealed that the microbial community changed depending on the nitrate loading rate and that high nitrate loading rates (low C_org_/NO_3_^-^) could lead to a diversification of anaerobic nitrate reduction toward the production of ammonium through DNRA, which contradicts to textbook knowledge claiming that DNRA should be favored under high C_org_/NO_3_^-^ ratios. Moreover, it was elucidated that higher nitrate loading rates can increase methane emissions in WC based denitrification beds.

The effect of nitrate loading rates on DNRA and methane emissions was unexpected and also highly unwanted regarding the desired application of these systems as sustainable and ecosystem-friendly nitrate elimination technology. The aim of this study was to elucidate whether this effect of high nitrate loading rates on the fate of carbon and nitrogen is specific to denitrification beds based on WC or whether other sustainable stable organic carbon materials would lead to the development of different microbial communities that would consequently also respond differently to variations in the nitrate loading rate. We expected the result of this study to be generic with regard to the suitability of a carbon source for the elimination of nitrate in a specific concentration window.

Our motivation is the planned construction of one of the first field denitrification beds in middle Europe, which is supposed to eliminate nitrate from agricultural drainage waters that run directly into a fen. The construction of such a field denitrification system at this but also at every other field site necessitates knowledge regarding the nitrate elimination rates and the resilience of the system toward different nitrate concentrations. Therefore, we show here the results from a 200-day laboratory triplicate experiments with wood pellets (WPs) and wheat straw as denitrification substrates and aim to correlate the results to the microbial community in the reactors and to our previous study with WC. Our results reveal that all three materials lead to unique microbial community compositions and sustain also individual responses toward nitrate pulses.

## Materials and Methods

### Laboratory Reactors and Analytical Analysis

The laboratory setup of the denitrification reactors was analogous to a former study with poplar WC as carbon source (Grießmeier et al., 2017). The WC had mainly a diameter particle size of 11–16 mm. This time the triplicates of the reactors were filled with either wheat straw or WPs (mixture of soft- and hardwood). The bed width in the reactors was 4 cm for both carbon sources, which corresponds to 30 g of wheat straw and 150 g of WPs. The volume of the WPs increased as soon as they were in contact with the liquid media. The swelling of the WPs was the reason why a lower amount of this substrate was added to the reactor compared to the WC (200 g). Only 30 g of wheat straw could be used due to low weight and the high volume. Autoclaved stones prevented the floating of the carbon substrates on the medium surface. Sample taking, nitrate addition, and the determination of CO_2_ and CH_4_ concentrations were conducted as described in Grießmeier et al. (2017). Also the used artificial moor media and inoculum were the same as in the previous study. The reactors were incubated at room temperature (approx. 22°C).

The starting concentration of nitrate was 1.18 mmol L^-1^, which correlated to the highest measured nitrate concentration of an agricultural drainage from a field site – a fen in the Vulkaneifel (Germany) – that we observe regarding the eutrophication effects of agricultural drainage waters. This fen is surrounded by agricultural fields and drainages lead directly into this sensible ecosystem.

In the experiment runtime of 200 days, samples were taken two to three times a week and analyzed for NO_3_^-^, NO_2_^-^, NH_4_^+^, and TOC according to Grießmeier et al. (2017) with a spectral photometer DR3900 and cuvette tests (Hach) as well as a TOC-analyzer (Multi N/C 2100 S Analytic Jena, Germany). Organic carbon compounds were analyzed via HPLC according to Kipf et al. (2013).

Whenever the nitrate concentration decreased below 0.24 mmol L^-1^, nitrate was added to reach the initial NO_3_^-^ concentration of 1.18 mmol L^-1^. The nitrate concentration was increased to the twofold (2.4 mM L^-1^), fivefold (6 mM L^-1^), 10-fold (12 mM L^-1^), and 20-fold (24 mM L^-1^) of the initial concentration to test the response of the systems to variations in the nitrate loading rates.

### DNA Extraction and 16S rRNA Gene Amplicon Sequencing

On day 70 all reactors of the triplicates were opened and samples were taken to analyze the biodiversity in the planktonic phase as well as in the biofilms growing on the surface of the organic carbon sources (WPs and wheat straw). Moreover, genomic DNA was extracted from drainage water from the study site (collected December 8, 2014) which served as inoculum.

Genomic DNA was extracted from 800 μl of the planktonic and from 200 to 300 mg of the solid samples using the innuSPEED Soil DNA Kit (Analytic Jena), as per the manufacturer’s guidelines.

The microbial diversity of each triplicate of the solid and the planktonic phase from all reactors as well as from the inoculum were analyzed via 16S rRNA gene amplicon sequencing. Sequencing was conducted by IMGM Laboratories GmbH (Martinsried, Germany) on an Illumina MiSeq platform using 2 × 250 bp paired-end (PE) reads. Moreover, to identify organisms that are involved in the denitrification process an amplicon sequencing on the Illumina MiSeq platform was performed targeting the functional gene *nirS*. Primer pairs used for the 16S rRNA amplicon sequencing were Bact_341F/Bact_805R for bacterial genes and A519F/A906R for archaeal genes (Stahl and Amann, 1991; Klindworth et al., 2013). Fragments of the *nirS* genes were amplified using primers cd3aF/R3cd (Throbäck et al., 2004).

### Statistical Methods and Bioinformatic Analysis

The maximum nitrate removal rate was determined using the maximum slope of the nitrate reduction kinetics for every reactor type. A one-way analysis of variance (ANOVA) and a following Tukey’s honestly significant difference (HSD) *post-hoc* test with a significance level of 0.05 was performed to analyze the statistical significance of the nitrate elimination rates sustained by the different carbon sources.

All bioinformatic analysis as well as the rarefaction curves and PCoA of the 16S rRNA gene amplicon sequencing were conducted as described previously with the CLC Genomic Workbench software 11.0.1 and the additional microbial genomic module 3.0. First all reads of every triplicate were quality trimmed with a limit of 0.05. Thereafter, a primer sequences trim and merging of the paired reads was performed. The OTU clustering was performed against the SILVA 16S v128 97% database with a similarity percentage specified by the OTU database and new creations were allowed with a taxonomy similarity of 80%. OTUs with a minimum combined abundance less than 50 were removed. OTUs belonging to Bacteria detected with the Archaea primer pair were excluded before the visualization of the relative abundance of the OTUs. Out of the triplicate of every sample the mean value for every OTUs was calculated. Alpha diversity was described with the phylogenetic diversity, the total number of OTUs and the Shannon index by aligning the OTUs using MUSCLE 2.0 and building a maximum likelihood based phylogenetic tree with the tree algorithm Neighbor Joining and nucleotide substitution model Jukes Cantor. A Principle Coordinate analysis (PCoA) was performed on the D_0.5 UniFrac distance.

For the analysis of the *nirS* gene amplicons the merged sequencing reads were analyzed using the blastN algorithm and a subset of sequences derived from the database of the European Nucleotide Archive (ENA) containing only nitrite reductases (E.C. 1.7.2.1, EC. 1.7.99.1) as target. Reads were accounted as *nirS* sequences if the gained *E*-value of the alignment was below 10^-10^. The phylogenetic assignment was conducted by grouping the hits with more than 100 reads of the blast analysis on the level of the bacterial order.

Supplementary Tables S1, S2 provides the results of the sequencing analysis parameters for each sample.

All raw reads of the amplicon sequencing that were retrieved for this study are publicly available through NCBI BioProject PRJNA445677 under SRA accession: SRP145155.

## Results and Discussion

### Nitrogen Species in the Denitrification Reactors

The objective of this study was to analyze the effect of different organic carbon sources in laboratory surrogates of environmental denitrification beds on the fate of nitrogen and carbon at varying nitrate loading rates and to analyze how this response would be catalyzed by the developing microbial communities. For more clarity, only the mean values are shown for all parameters. A detailed analysis of each triplicate is depicted in the Supplementary Figures S1–S4.

**FIGURE 1:**
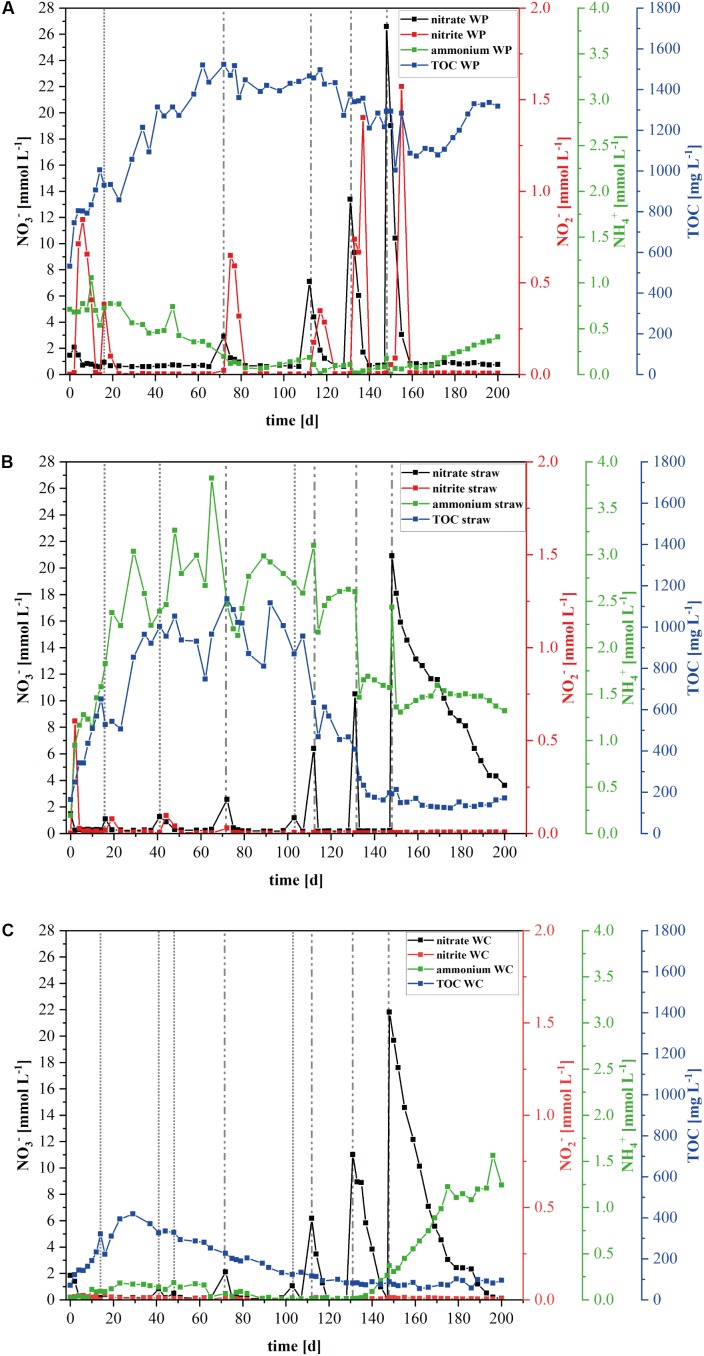
Development of the nitrogen species and total organic carbon (TOC) in the laboratory reactors. Mean value concentration of nitrate, nitrite, ammonium, and TOC in the laboratory denitrification reactors filled with **(A)** wood pellets (WP), **(B)** wheat straw, or **(C)** wood chips (WC). Data from the wood chip filled reactors derived from the earlier published investigation of Grießmeier et al. (2017). Dashed vertical lines represent the different nitrate addition events: dotted lines represent 1× (1.18 mmol L^-1^) nitrate addition, dotted and dashed lines represent 2× (2.36 mmol L^-1^) nitrate addition on Day 72, 5× (5.9 mmol L^-1^) nitrate addition on Day 112, 10× (11.8 mmol L^-1^) nitrate addition on Day 131, and 20× (23.6 mmol L^-1^) nitrate addition on Day 148.

Compared to WC as carbon source, the wheat straw and WP operated reactors were characterized by higher maximum contents of dissolved TOC (**Figure 1**). In fact, the TOC content in the WP reactors remained rather stable at 1.2 g/L although the nitrate concentration was raised up to 26 mM at day 148 of the experiment (**Figure 1A**). Moreover, the TOC components contained between days 40 and 150 on average 4.4 mM acetate, which is a readily degradable carbon source for a number of denitrifying model organisms. A detailed quantification of the detected organic acids is shown in the Supplementary Figures S5, S6. Since the TOC and the acetate concentration remained stable, it appears that denitrification was catalyzed independently of dissolved organic carbon and electron sources and might have been realized by a direct use of insoluble organic carbon in the form of cellulose by the denitrifying organisms. In contrary, the TOC content in the wheat straw reactors decreased drastically between days 89 and 137 which correlates to a nitrate addition of 1 mM at day 103 and 6.2 mM at day 112 (**Figure 1B**). Similar to the TOC, the mean acetate concentration also dropped within days 72 to 133 from 25 to 0.5 mM. This finding correlates well with other studies (Cameron and Schipper, 2010) that observed high effluent carbon leaching in the startup phase of barrels filled with wheat straw, which decreased over time. The WC filled reactors also showed a decrease in the TOC concentration, but this decrease started already at day 40 which also correlated with a decrease in the acetate concentration (**Figure 1C**). However, the mean maximum acetate concentration in these reactors was only 4.2 mM acetate (Supplementary Figure S5).

**FIGURE 2:**
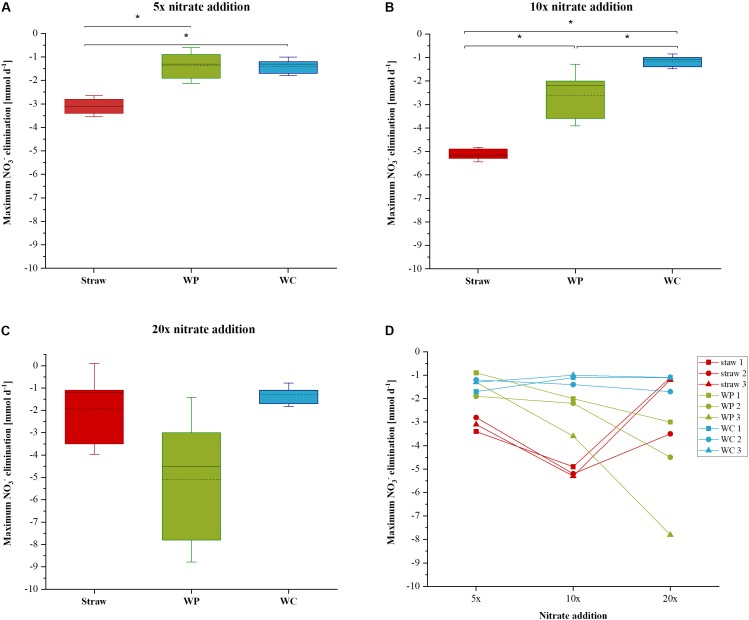
Maximum nitrate elimination rate for the 5× **(A)**, 10× **(B)**, and 20× **(C)** nitrate addition. Differences between the samples were determined by analysis of variance (ANOVA) followed by a Tukey *post-hoc* test. **(D)** Depicts the maximum nitrate elimination of each triplicate for every nitrate addition event. Significant differences (*p* < 0.05) between the samples are marked with an asterisks.

The nitrate elimination rates for moderate nitrate conditions were rather stable, especially in the wheat straw reactors. In the WP reactors the addition of nitrate to the initial concentration of 1.18 mM KNO_3_ was conducted only once, because these reactors could never lower the nitrate concentration below the threshold value of 0.24 mM, which was used in this study as the trigger for the addition of another nitrate pulse. The stable nitrate elimination behavior changed after addition of higher nitrate concentrations. Here, the nitrate elimination rates that were achieved with the three materials differed considerably both as a function of the initial nitrate concentration and of the carbon and electron source used. Especially the fivefold and 10-fold nitrate additions effectuated a significantly different behavior of the individual reactor types (**Table 1** and **Figure 2**). In the WP filled reactor the nitrate elimination rates increased with higher nitrate concentrations indicating that the system was limited by the concentration of the electron acceptor. In contrary, the WC system showed stable nitrate reduction rates almost irrespective of the added concentration. A third behavior occurred in the wheat straw reactors. Here, the 10-fold nitrate addition lead to a significant higher elimination rate, but decreased again considerably after addition of the 20-fold nitrate concentration, which might suggest a nitrate inhibition of the system (**Figure 2D**). The measured concentration of organic acids (acetate, propionate, butyrate, and fumarate) was very low at this time point in the wheat straw reactors. However, comparable low organic acid concentrations were also detected in the WC reactors and this did not affect the nitrate elimination rates to the same extent as in the wheat straw reactors (Supplementary Figures S5, S6).

**Table 1:** Maximal and mean nitrate elimination rate in each reactor type for the different nitrate addition events with additional *p*-values for the analysis of variance (ANOVA).

Reactor and carbon source	Maximum NO_3_^-^ elimination [mmol d^-1^]	Mean NO_3_^-^ elimination [mmol d^-^^1^]	ANOVA analysis *p*-value
**Fivefold (5.9 mM) NO_3_^-^ addition**			0.00186
Wheat straw 1	-3.4	-3.1 ± 0.3	
Wheat straw 2	-2.8		
Wheat straw 3	-3.1		
Wood pellets (WP) 1	-0.9	-1.4 ± 0.5	
Wood pellets (WP) 2	-1.9		
Wood pellets (WP) 3	-1.3		
Wood chips (WC) 1	-1.7	-1.4 ± 0.3	
Wood chips (WC) 2	-1.2		
Wood chips (WC) 3	-1.3		
**10-fold (11.8 mM) NO_3_^-^ addition**			0.00015
Wheat straw 1	-4.9	-5.1 ± 0.2	
Wheat straw 2	-5.2		
Wheat straw 3	-5.3		
Wood pellets (WP) 1	-2	-2.6 ± 0.9	
Wood pellets (WP) 2	-2.2		
Wood pellets (WP) 3	-3.6		
Wood chips (WC) 1	-1.1	-1.2 ± 0.2	
Wood chips (WC) 2	-1.4		
Wood chips (WC) 3	-1		
**20-fold (23.6 mM) NO_3_^-^ addition**			0.05991
Wheat straw 1	-1.1	-1.9 ± 1.4	
Wheat straw 2	-3.5		
Wheat straw 3	-1.2		
Wood pellets (WP) 1	-3	-5.1 ± 2.5	
Wood pellets (WP) 2	-4.5		
Wood pellets (WP) 3	-7.8		
Wood chips (WC) 1	-1.1	-1.3 ± 0.4	
Wood chips (WC) 2	-1.7		
Wood chips (WC) 3	-1.1		

The rather robust nitrate reduction in the WP systems went along with short pulses of nitrite that could be detected after every nitrate addition, no matter if at the beginning of the experiment – to reach the initial nitrate concentration - or in the later phase with higher nitrate concentrations (**Figure 1A** and Supplementary Figure S2). This indicates that the nitrate elimination was probably conducted by a different biocenosis compared to the other two systems.

The wheat straw reactors exhibited a high ammonium concentration over the whole-time course of the experiment (**Figure 1B** and Supplementary Figure S3). High start-up concentrations of ammonium for wheat straw reactors were also observed by Cameron and Schipper (2010). This might be a consequence of the higher initial ammonium concentration in the wheat straw biomass, compared to the other two carbon sources. What we could not observe was the nitrate triggered ammonium production which occurred in the WC reactors and was due to a change in the microbial community, especially an increase of organisms belonging to the order Ignavibacteriales (Grießmeier et al., 2017).

Constructed wetlands (CWs) offer another strategy to autonomously treat polluted or contaminated waters. The natural biocenoses of vascular and non-vascular plants together with a microbial community that resides in the root and stems that are in contact with the water can for instance catalyze the reduction of nitrogen contamination by the uptake via the plants and nitrification as well as denitrification. One difficulty for CWs is hydroponic wastewater (high nitrate, low organic carbon concentrations), because the plant material could be insufficient for heterotrophic denitrification (Park et al., 2015). Along these lines, typically a total nitrogen removal of around 40% was recorded for different CWs. Moreover, the typical mean inflow of total nitrogen reported for horizontal sub-surface flow CWs reported from different countries was around 46 mg l^-1^ and the mean inflow of NO_3_-N only 4.4 mg L^-1^ (Vymazal, 2007). This value is significantly lower than the starting concentration of NO_3_-N used in this study (74 mg l^-1^ NO_3_^-^/16.7 mg l^-1^ NO_3_-N). In contrary, CWs work rather well regarding the elimination of different pesticides and they might in fact be superior compared to denitrification beds regarding this application. Still, the removal of higher nitrate concentrations can most probably not be superior to denitrification beds as the latter provide a robust anoxic niche for denitrifying organisms and a constant supply of carbon and electrons.

### Microbial Community Composition

**FIGURE 3:**
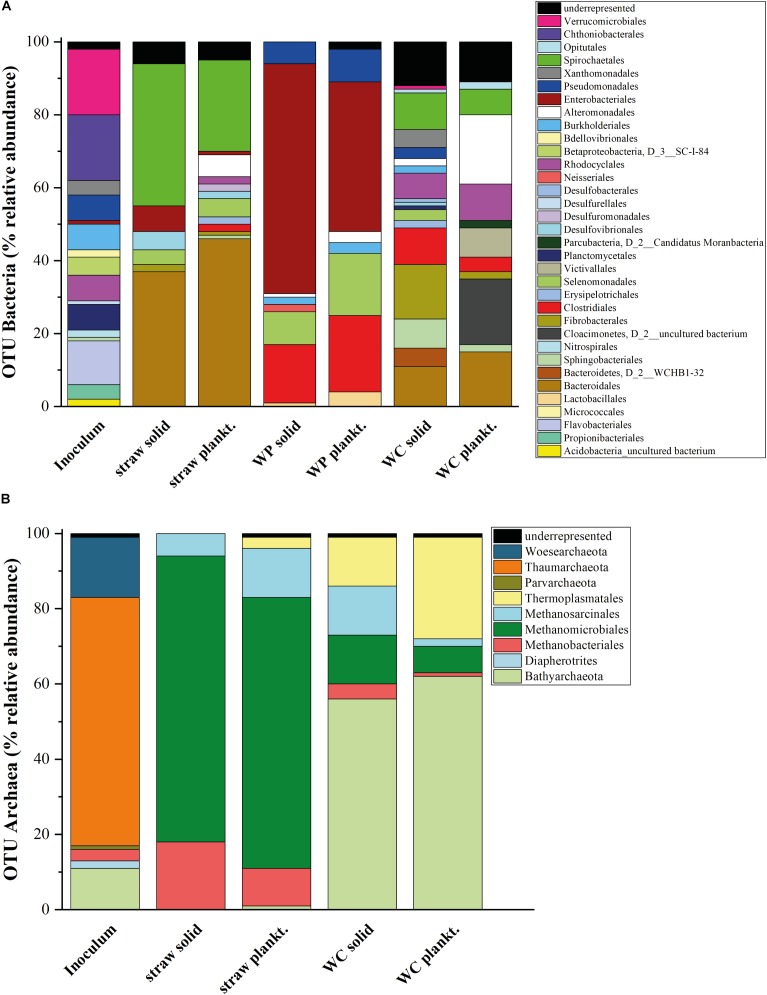
Microbial diversity in the laboratory reactors. Relative abundance of the 16S rDNA amplicon sequencing results for **(A)** OTUs of Bacteria (>1% of the total number of amplicons) and **(B)** OTUs of Archaea. SILVA 16S v 128 97% served as reference database. Results of the WC reactors were first published in Grießmeier et al. (2017).

Knowledge regarding the key players involved in nitrate elimination and cellulose hydrolysis in denitrification beds is very sparse. Thus, an amplicon sequencing of the 16S rRNA genes and the *nirS* genes with Illumina MiSeq was conducted to determine which members of Bacteria and Archaea might be involved in the denitrification process and if the carbon source leads to differing microbial compositions. All triplicates from each carbon source showed a comparable and repetitive composition among the triplicate samples (Supplementary Figure S7). The 16S rRNA gene based phylogeny displays a carbon source based succession of the initial inoculum toward three distinct microbial biocenoses (**Figure 3**). The alpha diversity (species richness) – here described with the phylogenetic diversity, total number of OTUs and the Shannon index (**Table 2** and Supplementary Figures S8–S10) - decreased in each sample in the order WC, wheat straw, WPs for the phylogenetic diversity, which might be due to the increasing homogeneity of the material. Especially the solid phase of the WC revealed the highest Shannon index closely followed by the inoculum. The Shannon entropy for the straw and WP reactors however showed comparable values for this diversity index. Moreover, also the total number of OTUs was higher in the WC reactors compared to the other two reactor types. The solid phase of the WC filled reactors in particular showed a higher Shannon entropy over their planktonic phase. Compared to that, the relative abundance and composition of bacterial OTUs was very similar in the planktonic and sessile phase of the wheat straw and WP reactors. In the reactors filled with straw, members of the order Bacteroidales dominated, mainly represented by organisms belonging to *Bacteroides* and *Microbacter.* Next to Bacteroidales, members of the order Spirochaetales dominated the straw filled reactors. Here, all organisms belong to the family Spirochaetaceae. The WP reactors instead showed a higher portion of members of the order Enterobacteriales (with the main representative *Enterobacter*), Clostridiales (with the main representatives *Caproiciproducens*, *Roseburia,* and *Lachnoclostridium*), Selenomonadales (main representative *Sporomusa*), and Pseudomonadales (main representative *Pseudomonas*). Members of the order Enterobacteriales and Clostridiales could also be found in the wheat straw reactors but at lower relative abundance. In the WP reactors, members of the order Bacteroidales and Spirochaetales were not detectable to a larger extent (**Figure 3A**). Especially members of the phyla Actinobacteria, Firmicutes and Proteobacteria, Bacteroidetes/Chlorobi are known to contain potential cellulose degrading organisms (Berlemont and Martiny, 2013), but cellulolytic activity was also detected in members of the Enterobacteriales (Rezaei et al., 2009). It seems that the hydrolytic cleavage of cellulose is dominated in WP and wheat straw filled reactors by different types of organisms. Of note, Clostridia were also revealed in the WC reactors to be participating in the cellulose degrading process (Grießmeier et al., 2017). As WPs are pressed sawdust, the similarity of the material might be the reason why the conditions selected in both reactor types for Clostridiales.

**Table 2:** Maximum values of different diversity indices – Shannon entropy, total number of OTUs and phylogenetic diversity – for each triplicate of the different samples as well as their mean value and standard deviation (SD).

Carbon source and phase	Shannon entropy		Total number of OTUs		Phylogenetic diversity	
			
	Max. value	Mean value/*SD*	Max. value	Mean value/*SD*	Max. value	Mean value/*SD*
Inoculum	5.6		103		2.4	
Wheat straw solid 1	4.5	4.1 ± 0.5	100	98 ± 13	5.2	4.6 ± 0.5
Wheat straw solid 2	4.3		110		4.5	
Wheat straw solid 3	3.5		84		4.2	
Wheat straw plankt. 1	4.8	4.5 ± 0.4	106	125 ± 22	5.4	5.3 ± 0.7
Wheat straw plankt. 2	4.5		149		6	
Wheat straw plankt. 3	4.1		119		4.6	
Wood pellets (WP) solid 1	4.7	4.2 ± 0.4	102	95 ± 14	2.6	2.2 ± 0.5
Wood pellets (WP) solid 2	3.9		79		1.6	
Wood pellets (WP) solid 3	4.1		104		2.4	
Wood pellets (WP) plankt. 1	4.8	4.5 ± 0.3	139	115 ± 23	2.4	3.3 ± 0.8
Wood pellets (WP) plankt. 2	4.1		113		3.8	
Wood pellets (WPs) plankt. 3	4.6		93		3.6	
Wood chips (WC) solid 1	5.5	5.8 ± 0.6	182	177 ± 6	6.3	6.4 ± 0.4
Wood chips (WC) solid 2	6.5		179		6.8	
Wood chips (WC) solid 3	5.4		171		6	
Wood chips (WC) plankt. 1	5.1	5 ± 0.1	183	187 ± 17	6.6	6.7 ± 0.4
Wood chips (WC) plankt. 2	4.9		205		7.1	
Wood chips (WC) plankt. 3	5.1		172		6.3	

The high relative abundance of members of the Enterobacteriales correlates well with the observed succession of nitrate reduction. Enterobacteriales are usually not denitrifying organisms but prefer the reduction of nitrate to nitrite first and reduce nitrite only after complete nitrate depletion (Tiso and Schechter, 2015). Recently, Lycus et al. (2017) used a new methodology to isolate nitrate reducing organisms from soil. The isolated Enterobacteriales could even reduce nitrate only to the level of nitrite or N_2_O. Hence, denitrification in the WP reactors could likely be catalyzed by two different phyla. The detected members of the Pseudomonadales (only from the genus *Pseudomonas*) and Burkholderiales (mainly belonging to the family Comamonadaceae) are two orders that are known to contain denitrifying organisms, which could either catalyze the full series of denitrification reactions or specialize on the reduction of nitrite produced by members of the Enterobacteriales (Lycus et al., 2017).

These two known denitrifying orders were accompanied in the WC reactors by members of the order Xanthomonadales (mainly from the genus *Pseudoxanthomonas*) which is also known to contain denitrifying organisms (Lycus et al., 2017) and could possibly also play an important role in the denitrification process. Interestingly, no order commonly known to contain denitrifiers was found in the solid phase of the wheat straw reactors. One possibility could be that organisms that were underrepresented (less than 1% relative abundance) may be responsible for the denitrification process here or are so far not known to be able to accomplish this process.

**FIGURE 4:**
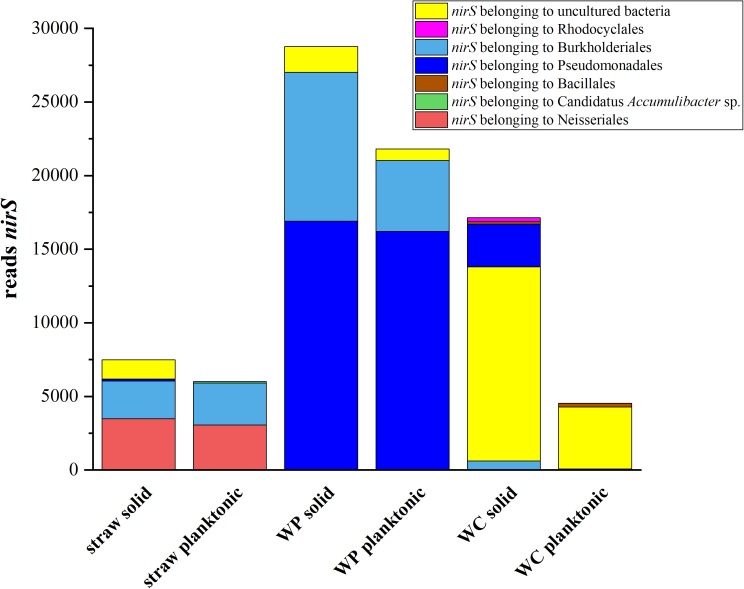
Total abundance of reads which could be assigned to *nirS* genes. Only nitrite reductases which inhabited an *E*-value lower than 10^-10^ and minimum read amount of 100 were used for further analysis.

To understand which organisms might be involved in the nitrate reduction process, an amplicon sequencing of the functional marker gene *nirS* was performed. The absolute amount of reads which could be assigned to the nitrite reductase gene differed considerably between the samples (**Figure 4**). However, a substantial difference in the composition of nitrite reductases could be found among the samples. The main part of nitrite reductases for the wheat straw reactors could be phylogenetically assigned to members of the order Neisseriales and Burkholderiales. Interestingly, the relative abundance of Neisseriales in the wheat straw reactor was only 0.02% and for Burkholderiales 0.1% for the solid and 0.5% for the planktonic phase. In the WP reactors most of the nitrite reductases could be assigned to members of the order Burkholderiales and Pseudomonadales. Both orders were also found in the 16S rRNA OTU dataset, but also here their abundance was with 2% (solid), 3% (plankt. phase) and 6% (solid), 9% (plankt. phase) rather low. Contrary to straw and WPs, most of the *nirS* sequences of the WC reactors could only be assigned to uncultured bacteria. A smaller fraction of reads could be assigned to Pseudomonadales and Rhodocyclales in the WC solid samples and both types of organisms were observed also in the 16S rRNA sequences. Still, for all reactor types the question remains whether this rather low concentration of organisms containing a *nirS* gene is sufficient for the observed nitrate elimination rates. Another possibility would be that we were unable to detect other denitrifiers of the community. The latter could be due to inefficient primer binding or because at least a part of the community uses the copper containing enzyme NirK instead of NirS. Still, our previous metatranscriptomic analysis of the WC reactors did not reveal sequences that could be assigned to *nirK* at low nitrate concentrations (Grießmeier et al., 2017). Nevertheless, Lycus et al. (2017) showed that many phylogenetic diverse organisms are engaged in the denitrification process and that a large proportion only performs a part of the whole denitrification process and showed a lack of genes for other denitrification reductases. Hence, division of labor could also be a reason why a small number of *nirS* encoding organisms could be sufficient to sustain the observed denitrification rates.

**FIGURE 5:**
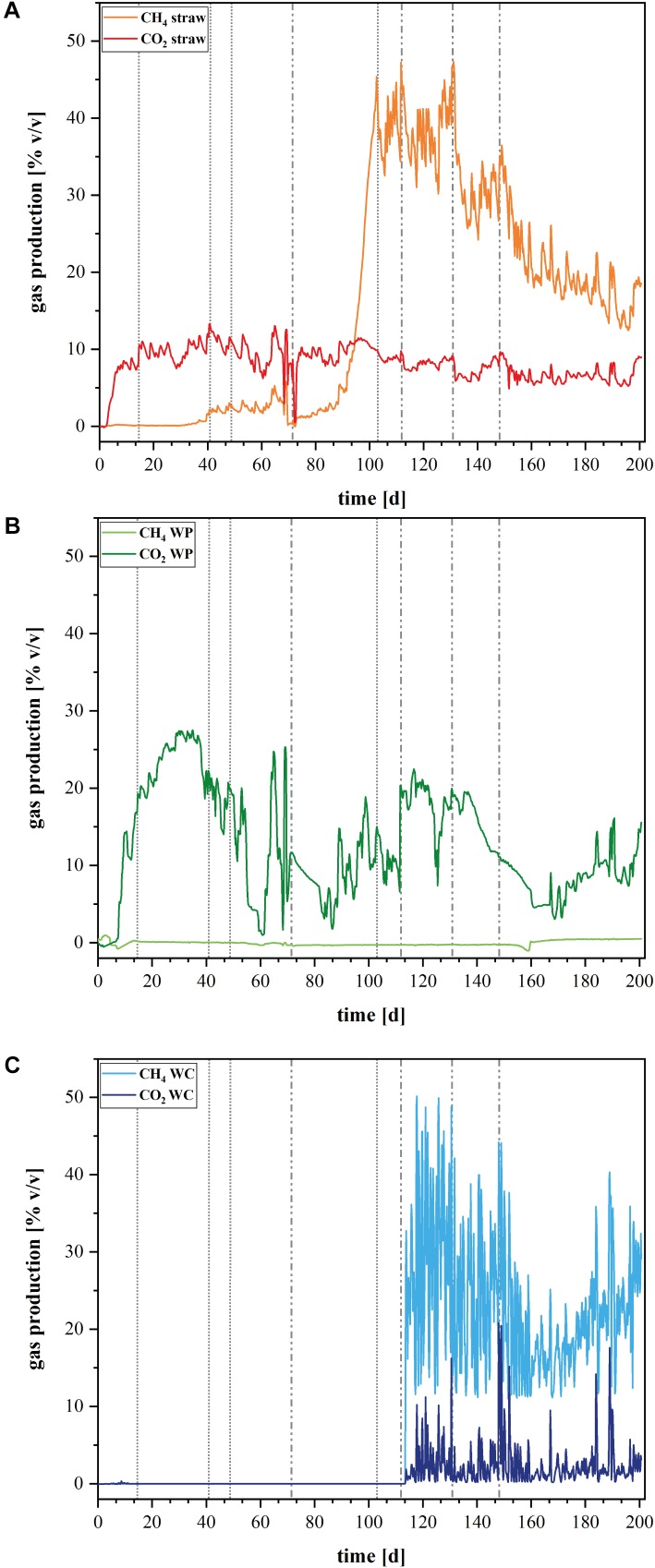
Gas production in the laboratory reactors. Volume percent [% v/v] of produced CH_4_ and CO_2_ in the different denitrification reactors **(A)** wheat straw, **(B)** wood pellets (WP), and **(C)** wood chips (WC). Dashed vertical lines represent the added nitrate concentrations: dotted lines represent 1× (1.18 mmol L**^-^**^1^) nitrate addition, dotted and dashed lines represent 2× (2.36 mmol L**^-^**^1^) nitrate addition on Day 72, 5× (5.9 mmol L**^-^**^1^) nitrate addition on Day 112, 10× (11.8 mmol L**^-^**^1^) nitrate addition on Day 131, and 20× (23.6 mmol L**^-^**^1^) nitrate addition on Day 148.

The conducted gas analysis corroborates the obtained phylogenetic data (**Figures 5A–C**). The archaeal OTUs in the straw reactors clustered only within members of methanogens, especially Methanomicrobiales. This correlates quite well with the methane production in these reactors, which increased considerably after day 90 (**Figure 5A**). The WC filled reactor contained next to methanogenic organisms, also Bathyarchaeota, organisms that are described as potential methylotrophic methanogens (Evans et al., 2015). It seems that these organisms could play a certain role for the methane cycle in these reactors. Compared to the other two reactor types, the WPs showed an extremely low outcome for the sequencing with the Archaea primer pair. None of the samples of the triplicate of the WP solid phase could be analyzed because the number of reads was too low and also two samples out of the triplicate of the planktonic phase of the WP sample revealed not enough reads. So only one sample out of the triplicate for the WPs planktonic phase could be used for analysis. An absolute amount of only 14 OTUs were identified here, which confirms that Archaea were highly underrepresented in these samples. Therefore, **Figure 3B** does not show data for the archaeal composition of the WP reactors. The lack or low number of Archaea correlates to the lacking methane production in these reactors (**Figure 5B**). So far, we do not know the reason for the inhibition of Archaea like methanogens in the WP reactors. WPs often contain varying amounts of heavy metals like for example zinc and copper in amounts of 1.8–12 mg kg^-1^ and 2.2–11 mg kg^-1^, respectively (Orecchio et al., 2016). Even if copper and zinc are also essential trace elements for hydrolytic microorganisms, Jarrell et al. (1987) showed in their study that these two elements can also have a toxic effect on different methanogenic strains even in the low concentrations that were quantified in WPs. Nevertheless, further elemental analysis of the WPs used in this study will show, if there is an increased heavy metal concentration, which could lead to the inhibition of methanogens.

## Conclusion

This study represents an enlargement of an earlier investigation (Grießmeier et al., 2017). The results of the nitrate elimination, gas production and microbial composition of denitrification reactors filled with either wheat straw or WPs were compared to this former study where WC were used as carbon source.

The results reveal that the choice of the carbon material not only determines the fate of the nitrate reduction, it also defines the microbial composition in the denitrification beds. The reactors filled with WPs seem to be suitable for high nitrate concentrations. Moreover, we could not measure methane emissions even at high TOC concentrations and over a time period of 200 days. Nevertheless, the occurring nitrite peaks and the gel like character of the soaked WPs are disadvantages of this material. The reactors with straw showed the best nitrate elimination rates for moderate nitrate concentrations. Still this material does not seem to be suitable for high nitrate loading rates. Moreover, high methane production and ammonia concentrations of almost 4 mM make this carbon source also unsuitable for a field denitrification bed. The clearest advantage of the WC over the other two materials seems to be the highly diverse microbial community that developed over time. The more diverse an ecosystem is the more robust it will be regarding process perturbations. These perturbations are system immanent, as the nitrate inflow, the temperature and the water flow through the systems in field scale compared to lab scale is hard to predict. In the future, we will be able to judge on this hypothesis by the analysis of field system that is supposed to rescue a fen in the Vulkaneifel in Germany.

## Data Availability

The raw data supporting the conclusions of this manuscript will be made available by the authors, without undue reservation, to any qualified researcher.

## Author Contributions

VG performed the experimental work including reactor design, analytical methods, DNA isolation, and bioinformatic evaluation of the 16S rRNA amplicon sequencing data, wrote the manuscript, and prepared all figures of the manuscript. JG supervised the project.

## Conflict of Interest Statement

The authors declare that the research was conducted in the absence of any commercial or financial relationships that could be construed as a potential conflict of interest.
