# Supplementary material for: Influence of the Potential Carbon Sources for Field Denitrification Beds on Their Microbial Diversity and the Fate of Carbon and Nitrate
Source: Front Microbiol. 2018 Jun 22;9:1313. doi: 10.3389/fmicb.2018.01313 (PMC6023987; doi:10.3389/fmicb.2018.01313)
Supplement: Supplementary file 1 [file Data_Sheet_1.docx]

Supplementary Material

Influence of the potential carbon sources for field denitrification beds on their microbial diversity and the fate of carbon and nitrate

Victoria Grießmeier, Johannes Gescher*

*** Correspondence:** Corresponding Author: Johannes.gescher@kit.edu

# Supplementary Figures and Tables

Table S1: Number of raw, merged and trimmed reads and number of reads in OTUs after phylogenetic analysis. SILVA 16S v 128 97% was used as reference database.

| **Sample** | **Raw reads** | **No. of reads after trim** | **No. of merged reads** | **Avg. length after trim** | **Reads in OTUs** |
| --- | --- | --- | --- | --- | --- |
| Inoc_Bac | 248 562 | 239 658 | 172 828 | 404.0 | 18 188 |
| Wheat straw solid 1_Bac | 23 200 | 23 177 | 22 794 | 404.0 | 3 537 |
| Wheat straw solid 2_Bac | 37 724 | 37 711 | 37 122 | 404.0 | 6 407 |
| Wheat straw solid 3_Bac | 38 294 | 38 260 | 37 220 | 404.0 | 4 840 |
| Wheat straw plankt. 1 _Bac | 32 286 | 32 194 | 31 630 | 404.0 | 5 412 |
| Wheat straw plankt. 2 _Bac | 67 094 | 66 986 | 65 980 | 404.0 | 12 108 |
| Wheat straw plankt. 3 _Bac | 46 792 | 46 744 | 45 840 | 404.0 | 8 092 |
| WP solid 1_Bac | 28 706 | 28 704 | 28 238 | 404.0 | 4 534 |
| WP solid 2_Bac | 36 598 | 36 593 | 36 142 | 404.0 | 10 981 |
| WP solid 3_Bac | 40 494 | 40 479 | 39 930 | 404.0 | 9 087 |
| WP plankt. 1_Bac | 129 642 | 129 471 | 127 946 | 404.0 | 25 550 |
| WP plankt. 2_Bac | 90 266 | 90 114 | 89 000 | 404.0 | 24 811 |
| WP plankt. 3_Bac | 37 482 | 37 456 | 36 864 | 404.0 | 6 508 |
| Inoc_Arch | 304 034 | 259 093 | 50 964 | 337.0 | 15 838 |
| Wheat straw solid 1_Arch | 25 872 | 25 868 | 23 672 | 337.0 | 11 156 |
| Wheat straw solid 2_Arch | 32 218 | 32 210 | 23 508 | 337.0 | 10 986 |
| Wheat straw solid 3_Arch | 44 336 | 44 334 | 40 638 | 337.0 | 19 035 |
| Wheat straw plankt. 1_Arch | 20 810 | 20 806 | 19 572 | 337.0 | 9 000 |
| Wheat straw plankt. 2_Arch | 17 202 | 17 202 | 16 242 | 337.0 | 7 634 |
| Wheat straw plankt. 3_Arch | 29 678 | 29 672 | 27 830 | 337.0 | 13 295 |
| WP solid 1_Arch | 17 224 | 17 224 | 1 376 | 337.0 | Number of reads too low |
| WP solid 2_Arch | 7 674 | 7 672 | 520 | 337.0 | Number of reads too low |
| WP solid 3_Arch | 12 290 | 12 290 | 6 670 | 337.0 | Number of reads too low |
| WP plankt. 1_Arch | 12 320 | 12 318 | 9 364 | 337.0 | 3 900 |
| WP plankt. 2_Arch | 6 978 | 6 978 | 5 634 | 337.0 | Number of reads too low |
| WP plankt. 3_Arch | 8 046 | 8 046 | 7 542 | 337.0 | Number of reads too low |

**Table S2:** Information on amplicon sequencing.

| **NCBI project ID** | PRJNA445677 |
| --- | --- |
| **SRA accession** | SRP145155 |
| **Taxonomic group** | Environmental sequencing project; Archaea and Bacteria |
| **Source of material A (drainage water)** |  |
| geographical location | Vulkaneifel (Germany) |
| latitude | 59° 9' N |
| longitude | 6° 53' E |
| depth | Surface water |
| time of sample collection | 2014-12-08 |
| sample type | Agricultural drainage water |
| **Source of the material B (enrichments in laboratory denitrification systems)** |  |
| geographical location | Karlsruhe Institute of Technology |
| sample type | Liquid and carbon material |
| time of sample collection | 2015-04-15 |
| habitat type | Agricultural drainage water |
| **Isolation and growth conditions** | Source material A: environmental sample  Source material B: Batch denitrification systems. Wheat straw and wood pellets served as carbon and electron source, nitrate as electron acceptor. Reactor were incubated in the dark at room temperature on a stirrer (125 rpm). |
| **Volume of samples** | 200-300 mg of wood pellets or wheat straw  0.8 ml of liquid sample |
| **DNA extraction method** | DNA isolation: InnuSPEED Soil DNA Kit, Analytic Jena |
| **Sequencing method** | DNA: amplicon sequencing (PCR with universal primer pairs Bact_541F/805R and A519F/U906R and nirS primer pairs cd3aF and R3cd); Illumina MiSeq, 2x250 bp |
| **16S rRNA gene analysis** | CLC Genomic Workbench software 11.0.1 with the additional microbial genomic module 3.0 |

## Supplementary Figures

**A**





**B**





**C**





**Supplementary Figure 1.** Nitrate concentration for each triplicate for the wheat straw reactors **(A),** the wood pellet reactors **(B)** and the wood chip reactors **(C).**

**A**





**B**





**C**





**Supplementary Figure 2.** Nitrite concentration for each triplicate for the wheat straw reactors **(A),** the wood pellet reactors **(B)** and the wood chip reactors **(C).**

**A**





**B**





**C**





**Supplementary Figure 3.** Ammonium concentration for each triplicate for the wheat straw reactors **(A),** the wood pellet reactors **(B)** and the wood chip reactors **(C).**

**A**





**B**





**C**





**Supplementary Figure 4.** Total organic carbon (TOC) concentration for each triplicate for the wheat straw reactors **(A),** the wood pellet reactors **(B)** and the wood chip reactors **(C).**

**A**


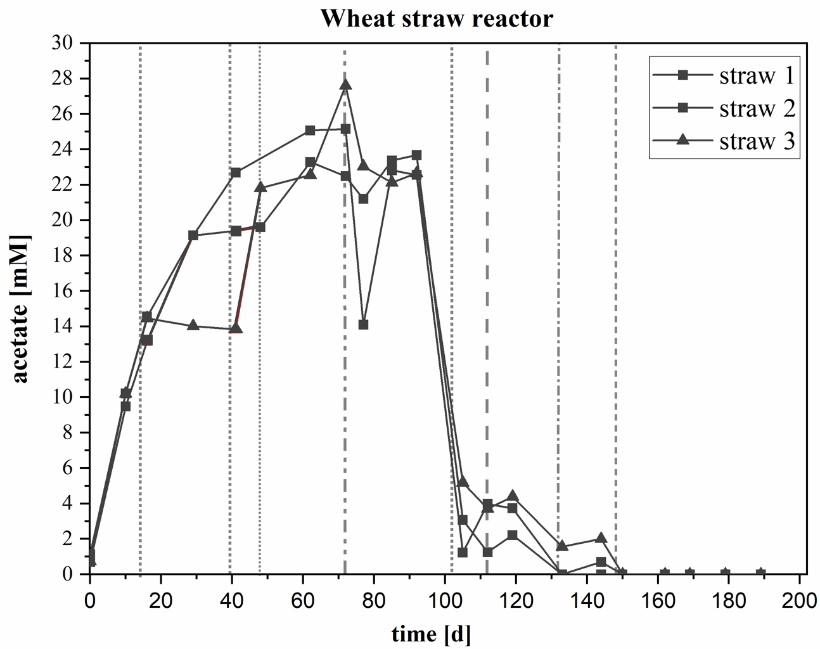


**B**





**C**





**Supplementary Figure 5.** Acetate concentration for each triplicate for the wheat straw reactors **(A),** the wood pellet reactors **(B)** and the wood chip reactors **(C).** Samples were analyzed every 10 to 14 days on the HPLC.

**A**





**B**





**C**





**Supplementary Figure 6.** Propionate concentration for each triplicate for the wheat straw reactors **(A),** the wood pellet reactors **(B)** and the wood chip reactors **(C).** Samples were analyzed every 10 to 14 days on the HPLC.


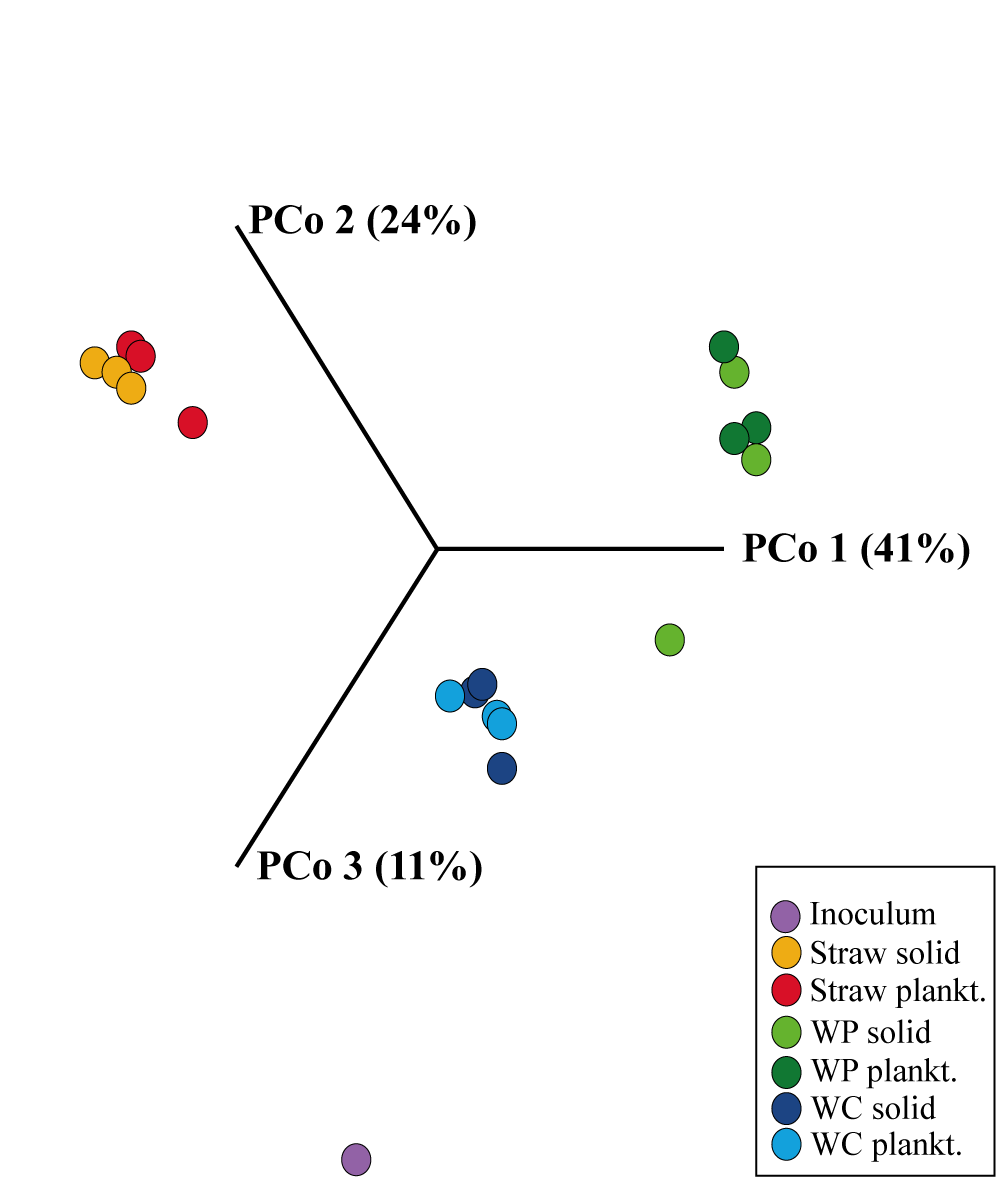


**Supplementary Figure 7.** PCoA analysis of all triplicates for the Inoculum (purple), straw solid (organge), straw plankt. (red), wood pellets solid (light green), wood pellets plankt. (dark green), wood chips solid (dark blue) and wood chips plankt. (light blue) sample.

**A**


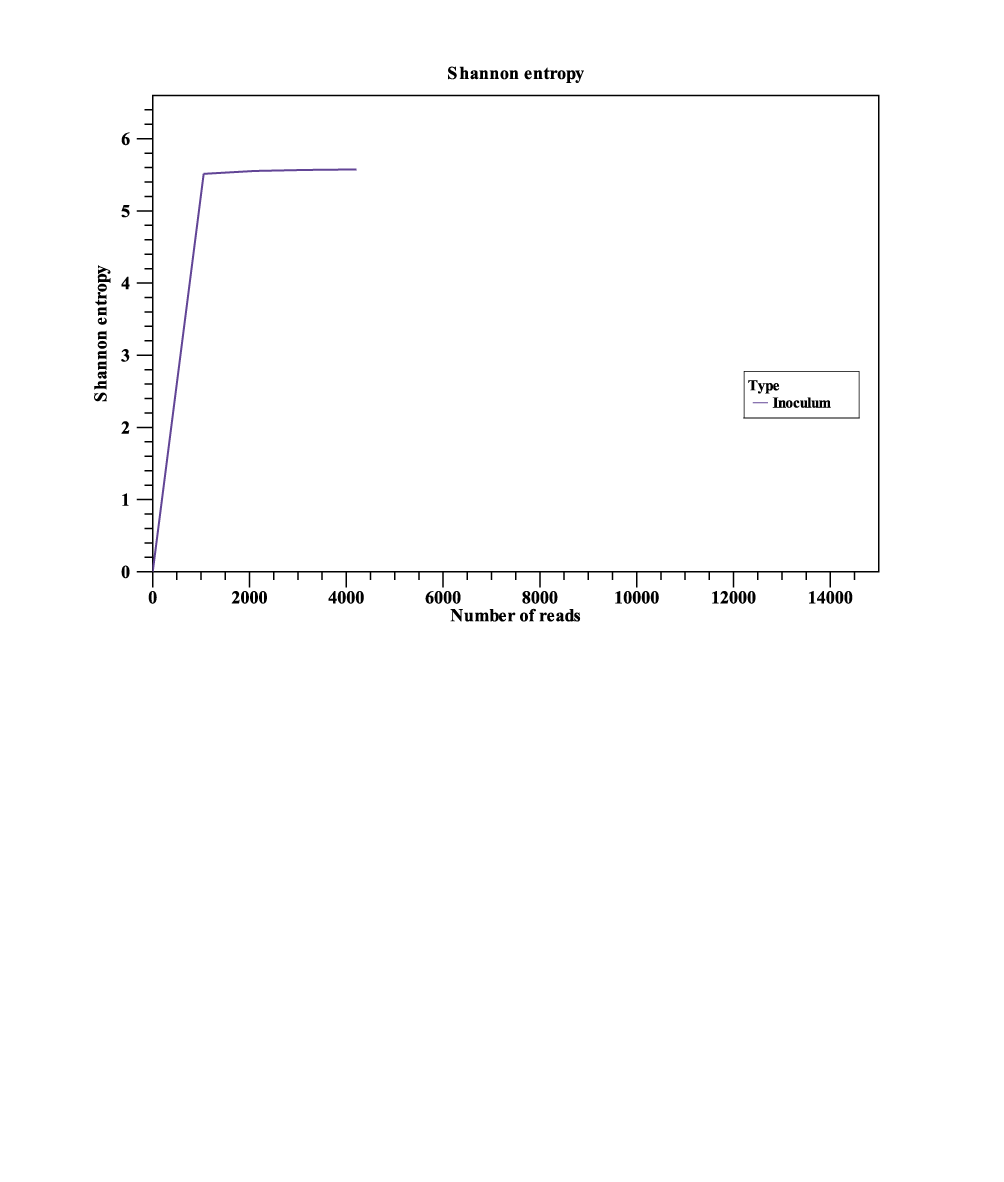


**B**


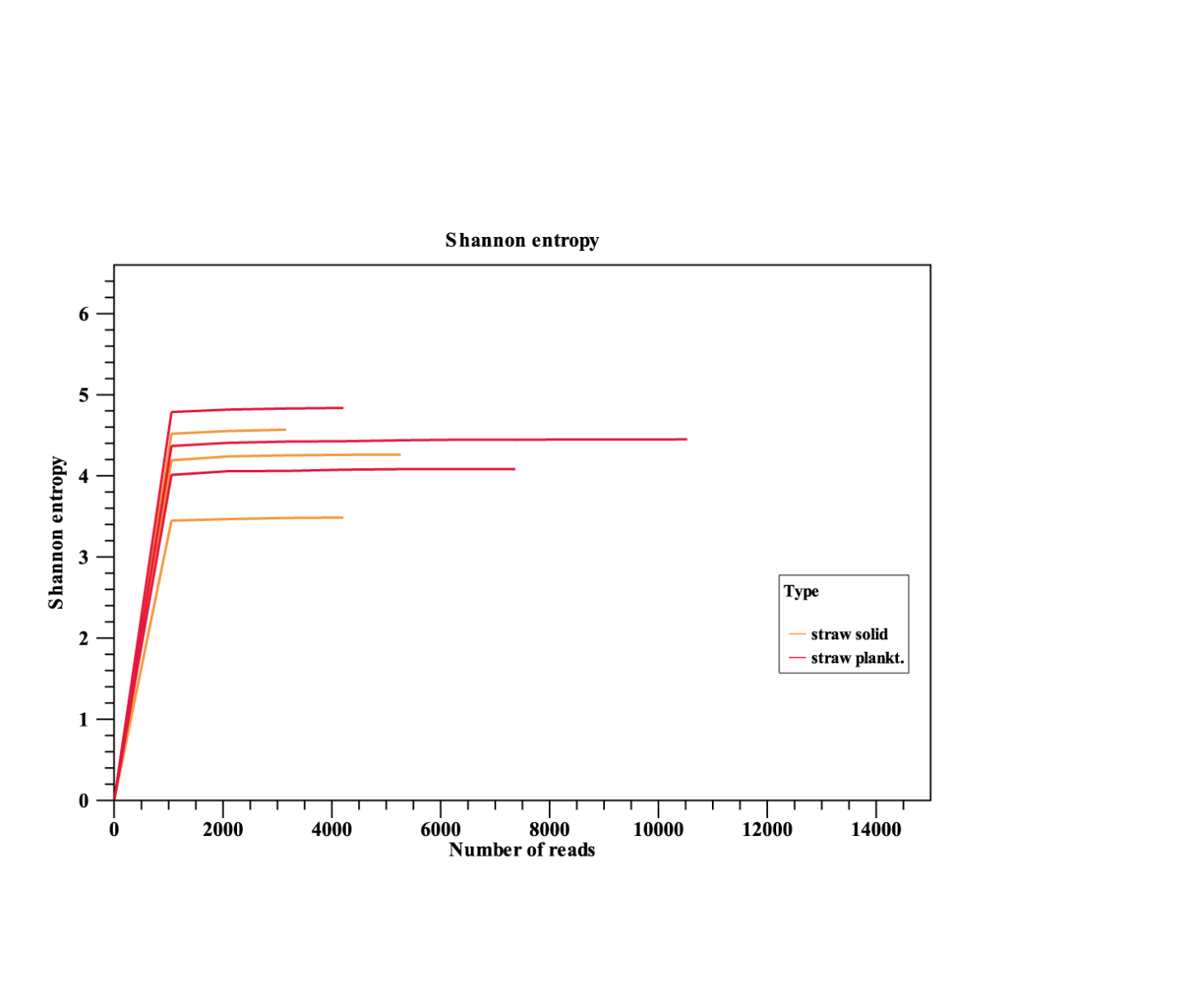


**C**


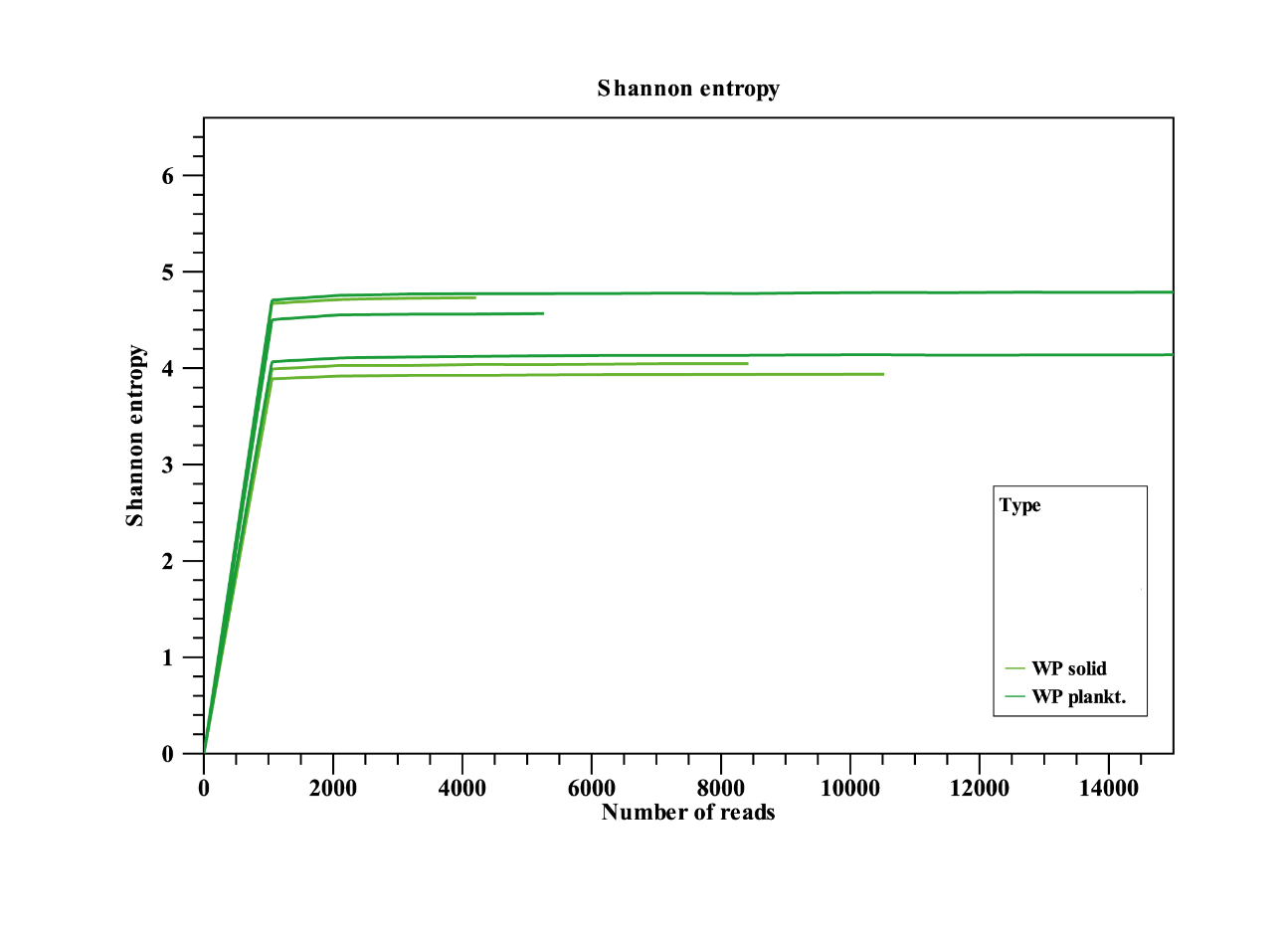


**D**


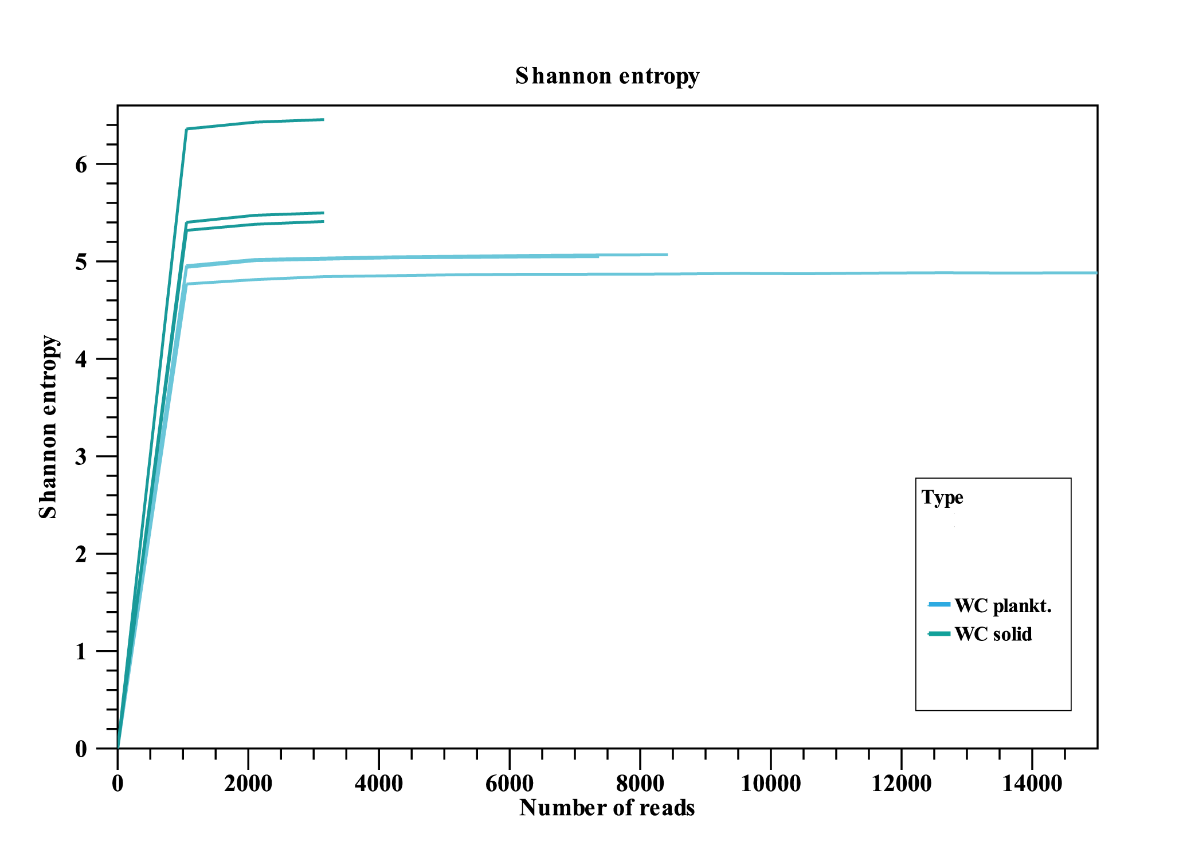


**Supplementary Figure 8.** Shannon entropy of the Inoculum **(A),** wheat straw reactors **(B),** wood pellet reactors **(C)** and wood chip reactors **(D).**


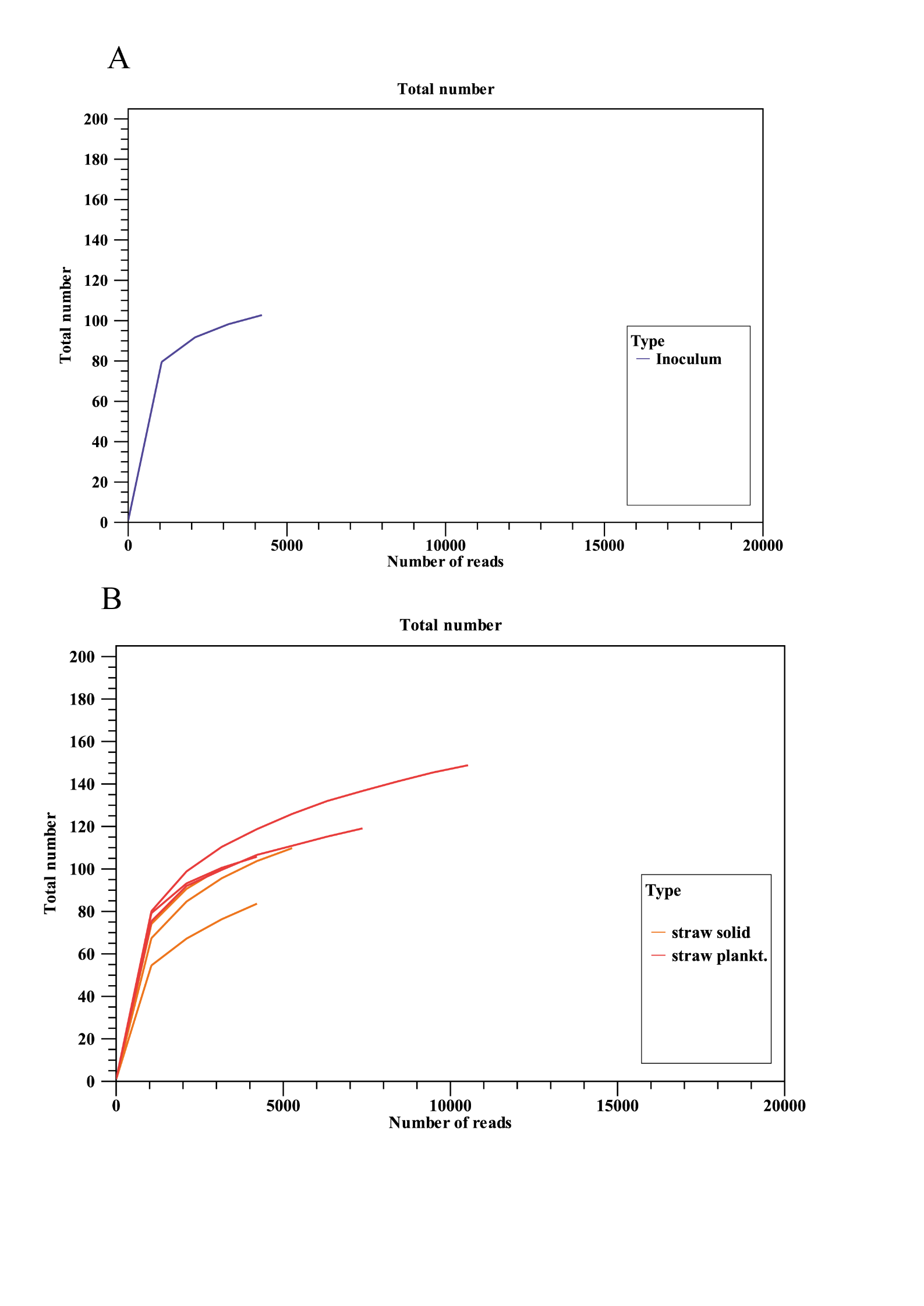


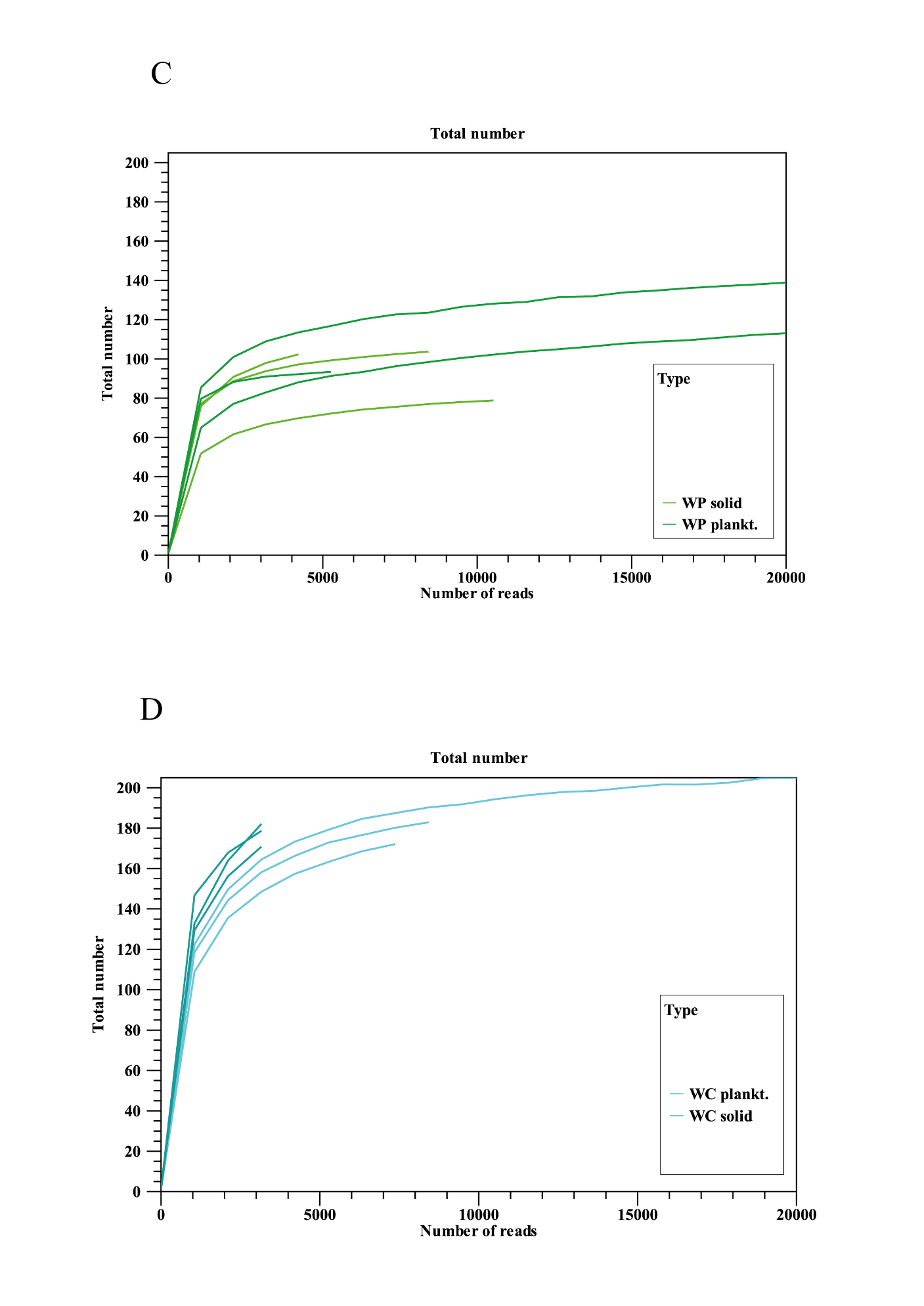
 **Supplementary Figure 9.** Total number of the OTUs of the Inoculum **(A),** wheat straw reactors **(B),** wood pellet reactors **(C)** and wood chip reactors **(D).**


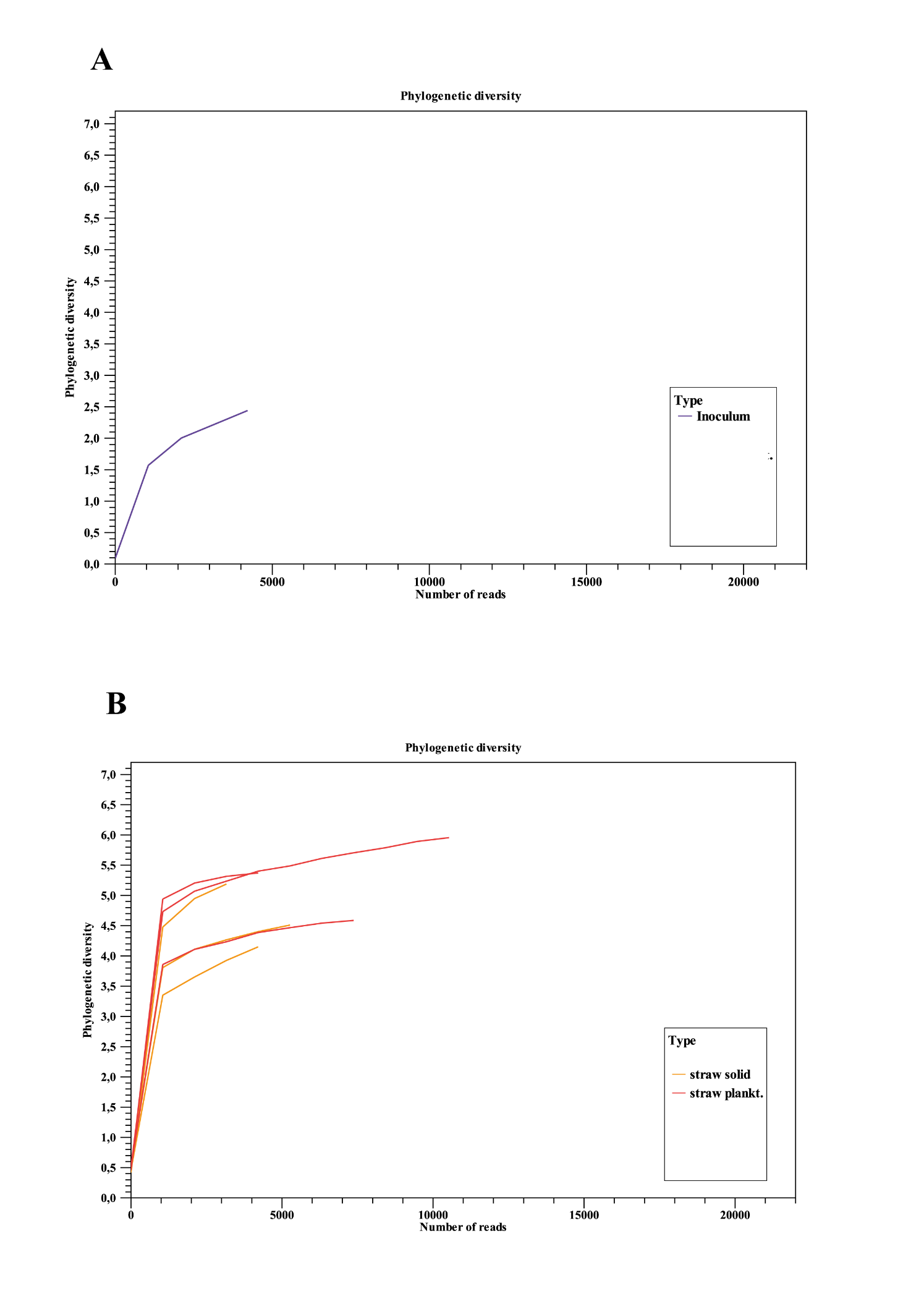


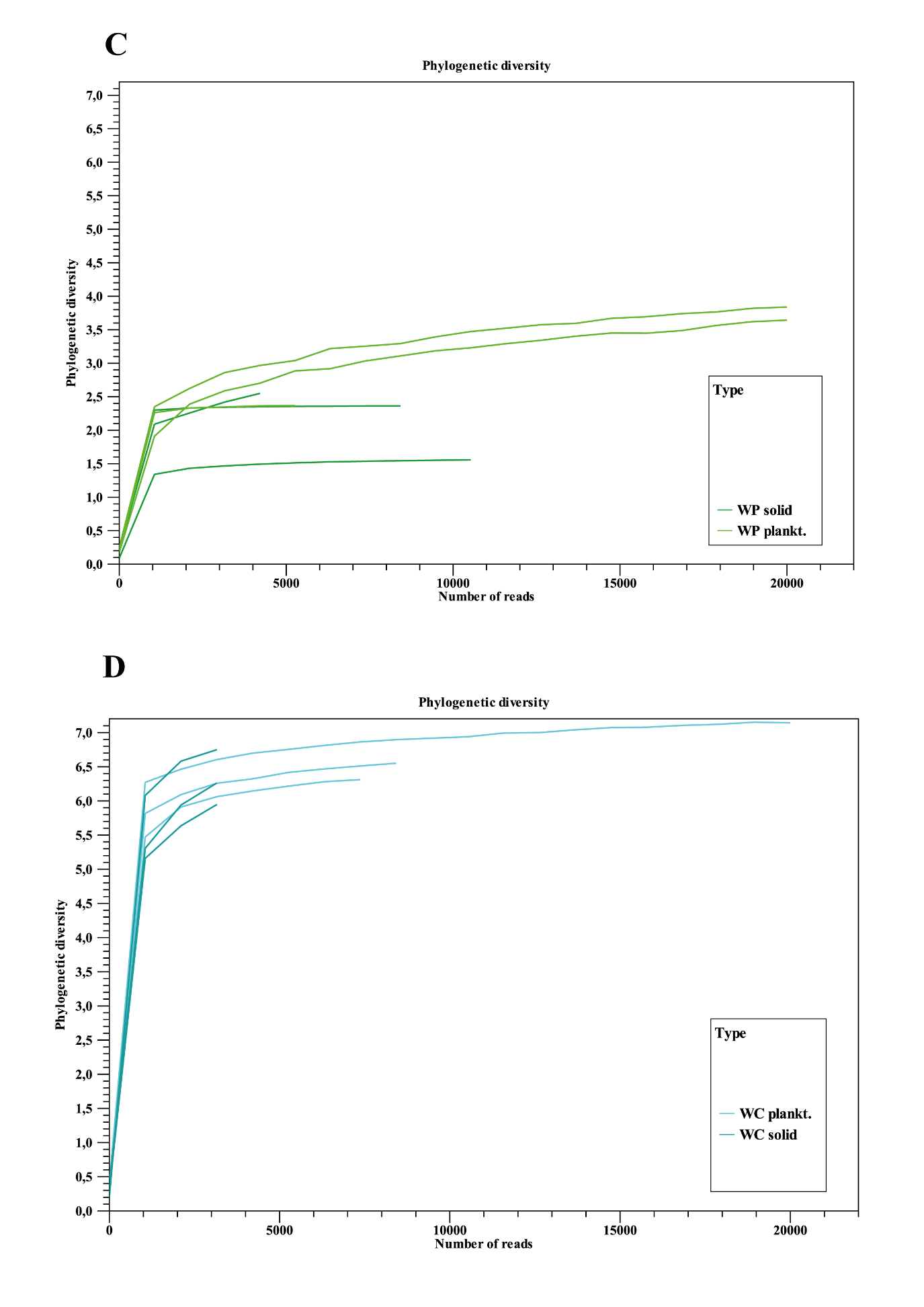


**Supplementary Figure 10.** Phylogenetic diversity of the Inoculum **(A),** wheat straw reactors **(B),** wood pellet reactors **(C)** and wood chip reactors **(D).**
